# Optimizing the catalytic activities of methanol and thermotolerant *Kocuria flava* lipases for biodiesel production from cooking oil wastes

**DOI:** 10.1038/s41598-021-93023-z

**Published:** 2021-07-01

**Authors:** Azhar Najjar, Elhagag Ahmed Hassan, Nidal Zabermawi, Saber H. Saber, Leena H. Bajrai, Mohammed S. Almuhayawi, Turki S. Abujamel, Saad B. Almasaudi, Leena E. Azhar, Mohammed Moulay, Steve Harakeh

**Affiliations:** 1 Department of Biology, Faculty of Science, King Abdulaziz University, Jeddah, Saudi Arabia; 2 Department of Botany and Microbiology, Faculty of Science, Assiut University, Assiut, Egypt; 3 Laboratory of Molecular Cell Biology, Department of Zoology, Faculty of Science, Assiut University, Assiut, Egypt; 4 Biochemistry Department, Faculty of Science, King Abdulaziz University, Jeddah, Saudi Arabia; 5 Department of Medical Microbiology/Parasitology and Molecular Microbiology Laboratory, King Abdulaziz University Hospital, Jeddah, Saudi Arabia; 6 Department of Medical Laboratory Technology, Faculty of Applied Medical Sciences, King Abdulaziz University, Jeddah, Saudi Arabia; 7 Vaccines and Immunotherapy Unit, King Fahd Medical Research Center, King Abdulaziz University, Jeddah, Saudi Arabia; 8 Preventive Medicine, General Directorate of Health Affairs, Aseer Region, Abha, Saudi Arabia; 9 Embryonic Stem Cells Research Unit, King Fahd Medical Research Center, King Abdulaziz University, Jeddah, Saudi Arabia; 10 Special Infectious Agents Unit, King Fahd Medical Research Center, King Abdulaziz University, Jeddah, Saudi Arabia; 11 Yousef Abdullatif Jameel Chair of Prophetic Medicine Application, Faculty of Medicine, King Abdulaziz University, Jeddah, Saudi Arabia

**Keywords:** Biotechnology, Microbiology, Environmental sciences

## Abstract

In this study, two highly thermotolerant and methanol-tolerant lipase-producing bacteria were isolated from cooking oil and they exhibited a high number of catalytic lipase activities recording 18.65 ± 0.68 U/mL and 13.14 ± 0.03 U/mL, respectively. Bacterial isolates were identified according to phenotypic and genotypic 16S rRNA characterization as *Kocuria flava* ASU5 (MT919305) and *Bacillus circulans* ASU11 (MT919306). Lipases produced from *Kocuria flava* ASU5 showed the highest methanol tolerance, recording 98.4% relative activity as well as exhibited high thermostability and alkaline stability. Under the optimum conditions obtained from 3D plots of response surface methodology design, the *Kocuria flava* ASU5 biocatalyst exhibited an 83.08% yield of biodiesel at optimized reaction variables of, 60 ^○^C, pH value 8 and 1:2 oil/alcohol molar ratios in the reaction mixture. As well as, the obtained results showed the interactions of temperature/methanol were significant effects, whereas this was not noted in the case of temperature/pH and pH/methanol interactions. The obtained amount of biodiesel from cooking oil was 83.08%, which was analyzed by a GC/Ms profile. The produced biodiesel was confirmed by Fourier-transform infrared spectroscopy (FTIR) approaches showing an absorption band at 1743 cm^−1^, which is recognized for its absorption in the carbonyl group (C=O) which is characteristic of ester absorption. The energy content generated from biodiesel synthesized was estimated as 12,628.5 kJ/mol. Consequently, *Kocuria flava* MT919305 may provide promising thermostable, methanol-tolerant lipases, which may improve the economic feasibility and biotechnology of enzyme biocatalysis in the synthesis of value-added green chemicals.

## Introduction

**Figure 1:**
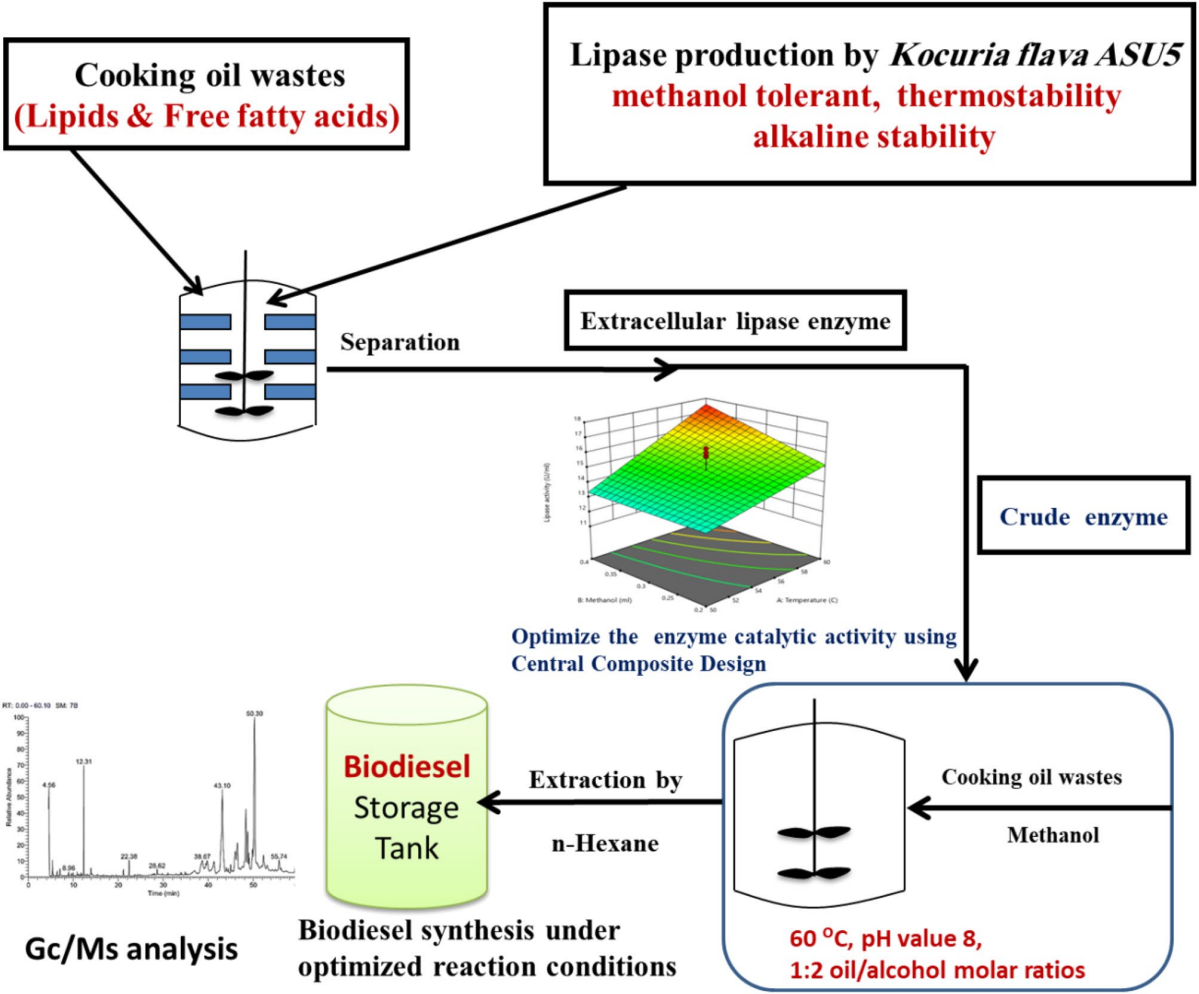
Graphical abstract showing overview of current work design.

In recent decades, due to the alarming increase in motorization, industrialization and human population; mankind faces the inevitable depletion in global strategic petroleum reserves on earth^1,2^. As well as the rapid increase in petroleum prices and alarming environmental crises makes bioenergy production from renewable raw materials is considered a sustainable alternative energy source for the future^3,4^. One of the most prominent potential renewable energy resources is biodiesel replacing the existing petroleum diesel due to its non-toxicity, eco-friendly and biodegradability^5^. Biodiesel is produced by transesterification/esterification methods through reacting fats/oils with short-chain alcohols in the presence of a homogenous chemical catalyst or solid heterogeneous chemical catalyst or enzyme biocatalysts^6–8^. The use of a homogenous chemical catalyst (KOH, NaOH, CH_3_ONa, HCl, H_3_PO_4_ and H_2_SO_4_) for chemical transesterification of lipids/fats has serious limitations, such as extensive downstream processing, the need for multiple purification steps, unused catalyst, soap formation and need for extensive wastewater treatment^9^. These drawbacks have gained significant attention in the search for different heterogeneous catalyst technologies for biodiesel synthesis. Recently, several studies stated solid basic or acidic heterogeneous catalysts for biodiesel production including, solid ferric hydrogen sulphate [Fe(HSO_4_)_3_]^10^, KF and NaNO_3_ catalysts^11^, Calcined dolomite^12^, Calcium lanthanum mixed oxide^13^, Bi_2_O_3_–La_2_O_3_^14^, calcium methoxide^15^, g-Al_2_O_3_/KI^16^, metal-doped methoxide^17^, sucrose-derived solid acid^18^, natural CaO^19^, sulfonated functionalized carbon^20^, ZrO_2_^21^, Methoxy-functionalized mesostructured stable carbon^22^, sand dollar^8^. The application of these heterogeneous catalysts for biodiesel production overwhelms the homogeneous catalysts drawbacks due to their feasibility, easily recovered processes, tolerance to feedstock moisture and free fatty acid contents and reusability^13^. So, the utilization of heterogeneous solid catalysts as an alternative to homogeneous chemical catalysts could lead to cost-competitive biodiesel production^7^. As well as, nanocatalysts revealed a high efficacy for biodiesel production from different lipid feedstock^23–26^. Furthermore, the enzymatic transesterification and esterification processes by microbial lipases may become an attractive alternative strategy for biodiesel production because of the reduced feedstock limitations, downstream processing steps, and environmental factors, in addition to the fact that lipase reactions do not form soaps and are not inhibited by water^27^. Lipases can completely convert free fatty acids in waste oil to fatty acid alkyl esters (FAAEs)^28^, which increase their potentiality and economic feasibility for biodiesel production. Although the enzyme transesterification and esterification approach is promising and has gained attention for the improvement of biodiesel production technology, there are some obstacles such as the inactivation of enzyme activity due to methanol^29^, high costs, and the complexity of enzyme purification. Therefore, it is crucial to improve and optimize reaction conditions to increase the catalytic activities and the feasibility of the enzymatic transesterification and esterification processes to yield a cost-effective and competitive biodiesel production technology, as shown with free-liquid enzymes^30,31^. Furthermore, immobilized lipases revealed enhancement of the catalytic activity of the reaction mixture and consequently improved the transesterification and esterification processes of both oils and free fatty acids^32–34^. Recently, several studies have been focused on the use of nanostructures including, nanofibers, nanocarbon and magnetic nanoparticles were for many biomedical applications^35^ as well as for supporting enzyme immobilization^36^. Sharma et al.^37^ investigated the efficacy of *Candida rugosa* lipase nanoparticles as a biocatalyst for biodiesel syntheses using free fatty acid-rich waste lipid sources (used cooking oil and brown grease). The obtained results from *Candida rugosa* lipase nanoparticles as a biocatalyst revealed the high performance of nanoparticles for biodiesel production which may be attributed to the inclusion of methyl-β-cyclodextrin subsequently their crosslinking and conjugation, which providing enhancement of lipase enzyme activity and stability^37^. Additionally, a new technology, based on ultrasound application, has been employed for the improvement of enzymatic transesterification and enzymatic esterification processes for biodiesel production that may enhance oil dissolution, increase conversion rate, and prompt protein conformational changes that may enhance enzymatic catalytic activities^38^. However, ultrasound may lead to lipase enzyme deactivation^39^ and consequently may cause inactivation of the enzymatic transesterification and enzymatic esterification processes and reduce the production yields of biodiesel. Moreover, this technology requires for enhancing the performance of low-energy consumption, high frequency piezoelectric ultrasonic reactor used to produce biodiesel from cooking oil wastes^40,41^ as well as specific bioreactors and infrastructure for large-scale biodiesel production. Conventionally, biodiesel can be produced in large quantities from virgin oils such as soybean, sunflower, corn, and cottonseed^32,42–44^. However, the high costs incurred in the production of these virgin oils make the produced biodiesel less competitive on the market as compared to petroleum diesel^45,46^. Moreover, the use of edible oils for biodiesel production gain serious concerns with global food security^46^. As a consequence of that, many researchers have worked on methods for biodiesel production from low-cost and available feedstock such as yellow and brown grease^47–49^, linseed^50^, castor^51^ and used cooked oils^52^. Non-edible oils and cooked oils contain high contents of free fatty acids (FFA) that can be converted to biodiesel via the esterification process. So, cooking oils are considered as a promising feedstock for biodiesel generation due to their availability in large quantities as food industrial wastes. Consequently, the current study focused on the potentiality of highly lipase producing thermotolerant bacteria for enzymatic transesterification and enzymatic esterification processes for biodiesel production from cooking oils wastes. These bacterial lipases were selected based on their methanol tolerance besides their thermotolerant activities. As well as the catalytic activities of the reaction conditions of bacterial lipases were improved for enhancement the efficacy of biodiesel production technology from cooking oil wastes as shown in Fig. 1. So, with the growing interest in biotechnological applications of lipolytic enzymes, our rationale in the current study was to (1) search for thermotolerant, highly lipase-producing bacteria having satisfactory properties to be used in an ongoing process for biodiesel production; (2) study and characterize thermostable and organic solvent-stable lipases from these thermotolerant bacterial species; (3) improve the catalytic activities and the potentiality of different thermostable, organic solvent-stable bacterial lipases to select the highly applicable bacterial lipases for biodiesel production from cooking oil waste; and (4) calculate the gross energy content of the biodiesel produced from cooking oil.

## Results and discussion

### Composition of cooking oil waste

**Figure 2:**
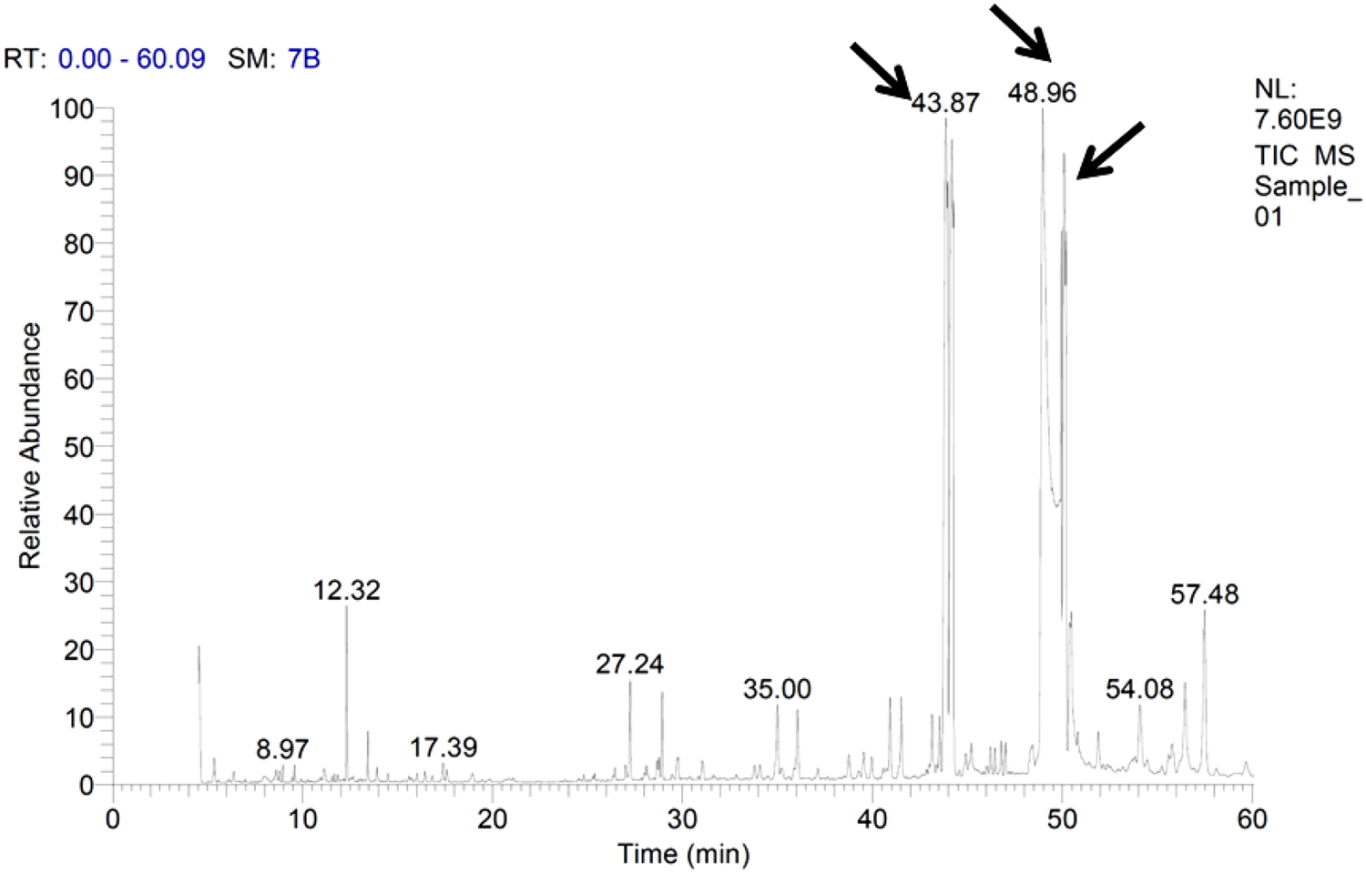
GC/MS profile of cooking oil waste composition and the labeled peaks represents the most common fatty acids.

Data obtained from the GC/MS analysis of cooking oil waste revealed that pentadecanoic acid, hexadecanoic acid, octadecanoic acid, and 9,12-octadecadienoic acid were the most common fatty acids recording 25.77, 24.49, 18.10, and 15.04% of total esters, respectively (Fig. 2). On the other hand, the other fatty acids were detected in the GC/MS profile in variable amounts; namely, methyl tetradecanoate, 9-hexadecenoic acid, cis-10-heptadecenoic acid, heptadecanoic acid, 11-octadecenoic acid, trans-13-octadecenoic acid, 6,9,12-octadecatrienoic acid, 11-eicosenoic acid, and nonadecanoic acid (Table 1).

**Table 1:** Composition of cooking oil waste.

Retention time (min)	Component	Value (% of total fatty acids)
36.06	Methyl tetradecanoate	1.29
43.53	9-Hexadecenoic acid	0.93
43.87	14-Methyl-pentadecanoic acid	25.77
44.18	Hexadecanoic acid	24.49
44.44	cis-10-Heptadecenoic acid	0.42
47.00	Heptadecanoic acid	1.08
48.95	9,12-Octadecadienoic acid	15.04
49.94	11-Octadecenoic acid	3.61
50.10	Octadecanoic acid	18.10
50.47	Trans-13-octadecenoic acid	1.67
51.89	7,10-Octadecadienoic acid	0.74
55.77	6,9,12-Octadecatrienoic acid	0.68
56.45	11-Eicosenoic acid	1.58
56.93	Linoleic acid	0.05
57.48	10-Methyl-nonadecanoic acid	3.54

### Isolation and screening for lipase-producing bacteria

Fifteen phenotypically thermotolerant bacterial isolates were recovered from cooking oil waste samples on an agar medium supplemented with cooking oil waste. Out of 15 bacterial isolates, 5 bacterial isolates revealed high lipolytic activity and were considered as high lipase producers. The highest production was recorded by ASU5 (16.04 U/mL ± 0.43 U/mL), followed by ASU15 (15.62 U/mL ± 1.83 U/mL), ASU10 (13.63 U/mL ± 0.53 U/mL), ASU11 (13.14 U/mL ± 0.03 U/mL), and ASU13 (12.08 U/mL ± 0.69 U/mL). The highest-lipase specific activity (215.52 U/mg protein) was estimated for ASU11 and followed by bacterial isolate ASU5 (106.93 U/mg protein), ASU10 (68.16 U/mg protein), ASU15 (56.17 U/mg protein), and ASU13 (53.28 U/mg protein), as shown in Table 2. Microbial lipases have drawn significant interest as of late for their applicability to a variety of potential biotechnological applications such as the synthesis of biodiesel, biopolymers, flavor compounds, agrochemicals, and green chemicals^53^. This is due to their unique properties such as their stability and activity in organic solvents; their substrate specificity; the fact that there is no requirement for enhancers or cofactors; and the fact that they display great enantio- and regioselectivity for the transformation of lipids and fats into fatty acids, ester, and glycerol, which may be easily recovered from the transesterification and esterification reactions^54^. These properties encourage the search for prospective applications of microbial lipases for the manufacturing of high value-added chemical products^54^. Industrial lipases are carried out by numerous microorganisms, including bacteria, filamentous fungi, yeasts, archaea, and actinomycetes, as well as the cultures of animals and plants. Among them, bacteria, fungi, and yeasts are considered promising candidates for the production of commercial lipases due to their great significance and the advantages of microbial lipases in many biotechnological processes such as more effective catalytic activities, high selectivity, require less energy consumption, work in mild conditions and environmentally friendly^55^. These could lead to the exponential development of microbial lipase biotechnology. On the other hand, the application for microbial lipases in the biodiesel industry showed many drawbacks including, lipase inactivation by methanol acyl acceptor, lipase stability, downstream processes and reusability^55^. Interestingly, the enzymes from thermophiles and thermotolerant microbes are known to be much more stable than those from mesophiles. Furthermore, these enzymes can maintain their activity for prolonged times, suggesting that they are more amenable to industrial applications^56^. So, the thermostable lipases with stability in organic solvent environments represent a significant advantage in biodiesel production^57^ because these solvents can facilitate the recovery of nonpolar products, enhance oil/fat solubility, and reduce the by-products in the organic solvent–water two-phase system^58^. Therefore, the use of heat and organic solvent stable lipases have potential applications in the synthesis of biopolymers as well as in the biodiesel and pharmaceutical industries, as the substrate lipids have high melting points. Thermal stability, therefore, is crucial for lipases as biocatalysts in the transesterification and esterification process of cooking oil for biodiesel production^59^.

**Table 2:** Extracellular lipase production, extracellular protein, and lipase-specific activity of bacterial isolates.

Test	Extracellular lipase production (U/mL)	Extracellular protein (mg/mL)	Specific activity (U/mg protein)
**Bacterial isolate**			
ASU1	8.69 ± 1.16	1.55 ± 0.27	5.62
ASU2	5.00 ± 0.55	0.19 ± 0.03	25.99
ASU3	8.18 ± 0.49	0.55 ± 0.02	14.96
ASU4	2.21 ± 0.06	2.16 ± 0.60	1.02
ASU5	16.04 ± 0.43	0.15 ± 0.03	106.93
ASU6	3.09 ± 0.73	0.50 ± 0.02	6.15
ASU7	2.79 ± 0.44	0.06 ± 0.01	48.85
ASU8	0	0	0
ASU9	1.60 ± 0.35	0.41 ± 0.02	3.95
ASU10	13.63 ± 0.53	0.2 ± 0.02	68.16
ASU11	13.14 ± 0.03	0.06 ± 0.01	215.52
ASU12	0.73 ± 0.18	0.23 ± 0.01	3.13
ASU13	12.08 ± 0.69	0.23 ± 0.03	53.28
ASU14	6.38 ± 0.11	0.25 ± 0.02	25.56
ASU15	15.62 ± 1.83	0.28 ± 0.02	56.17

Each value represents the average of three replicates ± SD.

### Methanol tolerant bacterial isolates

**Figure 3:**
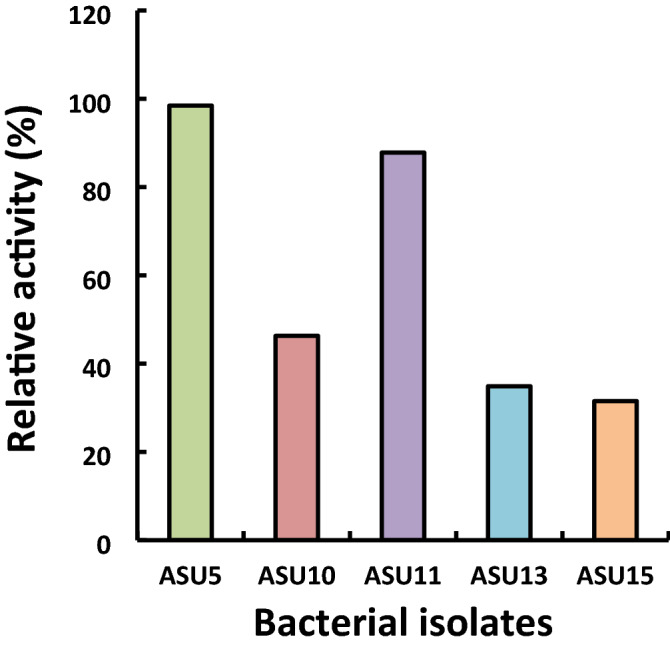
Effects of methanol on lipase enzyme produced by bacterial isolates.

The enzymatic transesterification processes for biodiesel production and alcohols show serious effects on the activity of the enzyme due to their inhibitory effect on lipases. To overwhelm this drawback, a stepwise addition technique involving methanol^60^ and organic solvents^61^ can be applied. Cervero et al.^62^ reported that a 90% biodiesel yield can be obtained by using a three-step addition method of alcohol, recording about 42% higher than the amount achieved using the one-step addition method. However, these methods are significantly time-consuming, as they boost the complications of experimental operations and have high operational costs on a large, industrial scale. Therefore, the search for novel, methanol-tolerant, lipase-producing isolates has become a new challenge. In this study, the highly lipase-producing isolates were screened for the selection of the potent lipase-producing bacteria with a high tolerance for methanol (acyl acceptor during the biodiesel production process). The results revealed that the highest tolerance for methanol by the tested bacterial isolates was recorded for bacterial isolate ASU5 (relative activity, 98.4%) followed by bacterial isolate ASU11 (relative activity, 87.8% [Fig. 3]). Consequently, bacterial isolates ASU5 and ASU11 were considered potent isolates for lipase production, with the most tolerant isolates for methanol, which were subsequently selected for optimizing the catalytic activities of lipase using the one-factor-at-a-time approach and the response surface methodology design.

### Phenotypic and phylogenetic identification of bacterial isolates

**Figure 4:**
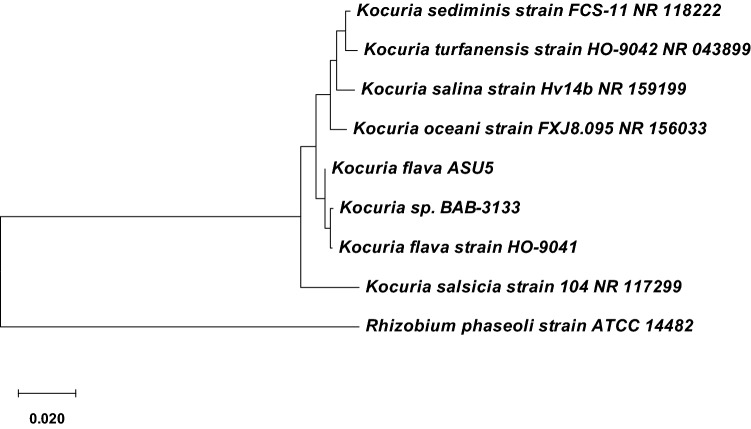
Phylogenetic tree of lipase-producing strain *Kocuria flava* ASU5 (MT919305).

**Figure 5:**
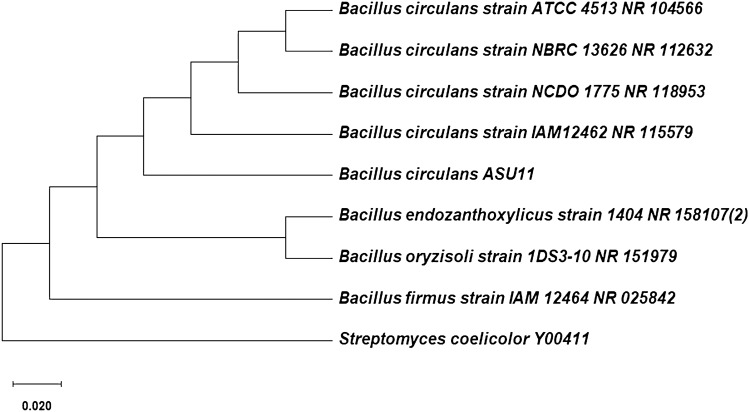
Phylogenetic tree of lipase producing strain *Bacillus circulans* ASU11 (MT919306).

Phenotypic characterization of the potent lipase-producing bacterial isolates with the highest tolerance for methanol was tentatively estimated for two different bacterial isolates (Table 3). The phenotypic identification was assessed by 16S rRNA gene phylogenetic analysis. The partial sequence of 891 base pairs of bacterial isolate ASU5 had sequence similarity with 99.89% similarity to the *Kocuria flava* strain HO-9041 (NR044308); whereas the 16S rRNA gene sequence of 905 base pairs for bacterial strain ASU11 exhibited 98.45% similarity to the *Bacillus circulans* strain ATCC 4513 (NR104566) and 97.57% similarity to the *Bacillus circulans* strain IAM12462 (NR115579). As a result, based on the phenotypic characterization (Table 3) and genotypic analysis of the bacterial strains, ASU5 and ASU11 were identified as *Kocuria flava* and *Bacillus circulans*, respectively, and their sequences were deposited under the accession numbers MT919305 and MT919306, respectively. A phylogenetic tree was assembled from 16S rRNA multiple sequences alignment (Figs. 4 and 5).

**Table 3:** Phenotypic characterization of methanol-tolerant and highly lipase-producing bacterial isolates.

Characteristics	Bacterial isolates	
	ASU5	ASU11
**Gram staining**	**+**	**+**
**Cells**	Coccoid	Rod-shaped
**Oxidase**	**−**	**−**
**Nitrate reductase**	**+**	**−**
**Catalase test**	**+**	**+**
**Gelatin hydrolysis**	**−**	**+**
**Casein hydrolysis**	**+**	**+**
**Starch hydrolysis**	**+**	**+**
**Indole production**	**−**	**−**
**Urease test**	**+**	**−**
**H**_**2**_**S production**	**−**	**−**
**Esculin test**	**−**	**+**
**Voges-Proskauer**	**−**	**−**
**Carbon source utilization:**		
l**-**Arabinose	**+**	**−**
d**-**Cellobiose	**−**	**+**
d**-**Fructose	**−**	**+**
Citrate	**−**	**−**
d**-**Alanine	**−**	**−**
d**-**Sorbitol	**+**	**+**
d**-**Galactose	**+**	**+**
Glycerol	**+**	**+**
Glucose	**+**	**+**
Lactose	**+**	**+**
Maltose	**+**	**+**
Mannitol	**+**	**+**
**Growth at 5 °C**	**−**	**−**
**Growth at 37 °C**	**+**	**+**
**Growth at 45 °C**	**+**	**+**

Microbial lipases are produced by several microbial species, including bacteria and fungi, such as *Pseudomonas fluorescens*^63^, *Burkholderia cepacia*^64^, *Staphylococcus haemolyticus*^65^, *Chromobacterium viscosum*^66^, *Phichia pastoris*^67^, *Mucor miehei*^68^, *Aspergillus terreus*^69^, *Rhizopus oryzae*^70^*, Candida cylindracea*^71^, and *Candida rugosa*^72^. The potentiality for bacterial lipase production is attributed to their ability to utilize inducers of lipids and fats such as vegetable oil, oil industry waste, cooking oil waste, surfactants, and triglycerides through hydrolytic processes by the inducible lipases^73^. Zhou et al.^74^ stated that *Kocuria flava*, Gram-positive bacteria, grow aerobically at 28 ^○^C to 45 ^○^C with a pH of 7 to 9; the cells are non-motile, coccoid cells. Additionally, *Kocuria flava* is characterized by positive reactions for nitrate reduction, catalase, amylase, and urease production, but negative results are exhibited for oxidase, gelatinase, and indole production. Furthermore, *Bacillus* is grown aerobically and it is Gram-positive with rod-shaped, endospore-forming cells^75^.

Abd-Alla et al.^76^ reported that *Bacillus vallismortis* ASU3 (KP777551), *Bacillus tequilensis* ASU11 (KP777550), *Bacillus amyloliquefaciens* ASU16 (KP777549), and *Bacillus firmus* ASU32 (KP777552) exhibited the highest lipase production, recording 4.72 U/mL, 3.13 U/mL, 3.41 U/mL, and 4.28 U/mL, respectively. *B. firmus* ASU32 (KP777552) showed the highest activity toward the lipase transesterification processes of fungal lipids and displayed higher thermal stability and methanol tolerance, expecting their application as a promising biocatalyst for fatty acid methyl ester (FAME) synthesis.

### Optimization of organic solvent stability, alkaline and thermostability of bacterial lipase activities

#### One-factor-at-a-time method

The impact of acyl acceptors on lipase activity as well as enzyme stability and specificity is considered a crucial factor in the biocatalysis of lipids for biodiesel production, due to their effects on lipases’ catalytic performances^77^. Unfortunately, this issue has only been studied from one point of view, without taking into consideration the combination of other factors of the reaction mixture on enzyme activity (temperature, pH, acyl acceptor, etc.) that are most effective in the hydrolysis process of interest^78^. Therefore, getting insights into the interaction effects of the enzyme activity by the experimental design is of vital interest, both for lipase application and for developing the knowledge obtained from the bases of the lipase kinetic and structural robustness or sensitivity of the enzyme under the studied factors^78^. So, this study aimed to study the effect of individual factors (acyl acceptors, acyl acceptor concentration, temperature, and pH value) of the reaction mixture on the catalytic activity of a bacterial lipase as well as the interactions of these factors on the catalytic process^79^.

##### Effect of different organic solvents on lipase activities

**Figure 6:**
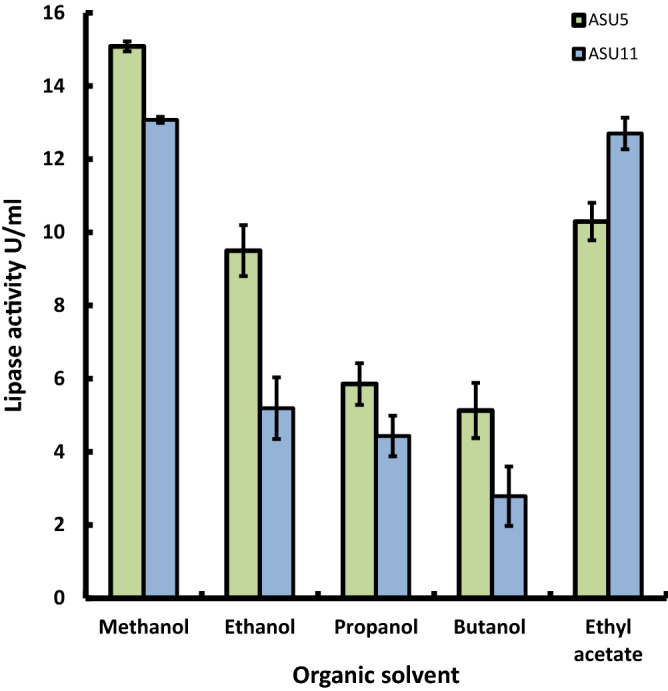
Effect of different acyl acceptors (0.1 mL) on bacterial lipase. The means value of three replicates ± SD (vertical bars) is represented.

Different organic solvents were assayed for their effect on lipase activities of the two highly lipase-producing, methanol-tolerant bacterial isolates. Data are shown in Fig. 6 revealed that among the acyl acceptors investigated, methanol and ethyl acetate was the less active acceptors of the activities of lipases. Consequently, these acyl acceptors were considered the most active organic solvents for biodiesel production from waste oil, compared to other acyl acceptors (e.g. ethanol, propanol, and butanol) due to the reduction of lipase activities. Alcohols are the common acyl acceptors, mostly methanol (MeOH) and ethanol (EtOH) in addition to propanol, isopropanol, butanol, and branched alcohols that are not preferred as acyl acceptors due to their high cost^80^. Methanol is the best choice for the transesterification process, rather than ethanol and ethyl acetate, because it is inexpensive and more reactive, in addition to the fact that the FAME is more volatile than the fatty acid ethyl esters (FAEE)^81^. FAME also has slightly low viscosity and high cloud and pour points compared to the corresponding FAEE^82^. So, acyl acceptors (methanol) are considered the most realistic options for large-scale biodiesel production compared to the other, more expensive acceptors (methyl acetate and ethyl acetate).

##### Effect of methanol concentration on lipase activities

**Figure 7:**
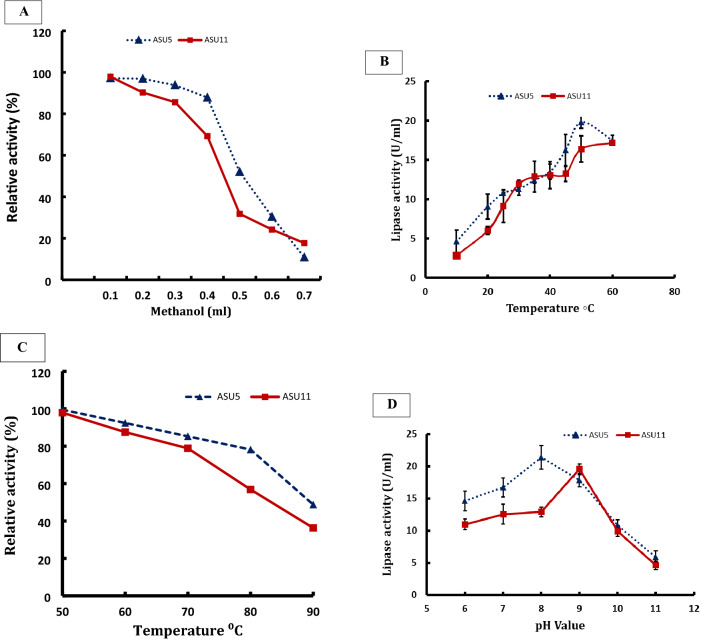
(**A**) Effect of methanol concentration on bacterial lipase activities. (**B**) Effect of temperature on bacterial lipase activities. The means value of three replicates ± SD (vertical bars) is represented. (**C**) Thermoactivity of lipases produced by bacterial strains *Kocuria flava* ASU5 and *Bacillus circulans* ASU11 (MT919306). (**D**) Effect of pH value on bacterial lipase activities. The means value of three replicates ± SD (vertical bars) is represented.

The transesterification process involves stepwise, reversible steps of a triglyceride with an acyl acceptor to form biodiesel, so a slight excess of acyl acceptor is used to alter the reaction toward biodiesel formation. The concentration of methanol, then, on the reaction mixture is a limiting factor for lipase enzyme activity. Consequently, the transesterification process for biodiesel production is inhibited due to the inhibitory effect on lipases^34^. Data in Fig. 7A show that methanol is a limiting factor for the catalysis of the reaction, as by increasing the concentration of methanol from 0.1 to 0.4 mL in the reaction mixture the activity of lipase is maintained, recording a relative activity of 87.84% for *Kocuria flava* ASU5 (MT919305), whereas the catalytic activity for *Bacillus circulans* ASU11 (MT919306) recorded a relative activity of 85.95% at methanol concentration 0.3 mL (Fig. 7A). Then, the activity decreased dramatically until it was fully inactivated. The results showed the potency of the bacterial strain *Kocuria flava* ASU5 (MT919305) lipase as a biocatalyst for biodiesel production, due to the tolerance mechanism compared to the bacterial strain *Bacillus circulans* ASU11 (MT919306). Methanol is sometimes used in biocatalysis processes to increase the solubility of reaction substrates; it is also used as an acyl acceptor in biodiesel production through lipase transesterification processes, but the inhibition of lipases is detected in several cases. That is why the impact of methanol on the catalytic activity and conformation of *Burkholderia glumae* lipase and *Candida antartica* lipases was investigated; these were recorded as highly methanol-tolerant^83^. Additionally, the inactivation effects were applied from the damage in enzyme stability due to the gradual protein unfolding and aggregation^83^. *Candida antartica* exhibited the highest lipase activity at methanol concentrations of 0.7%; after that, there was a sharp reduction at higher methanol concentrations. Consequently, a thermodynamic model of *Candida antartica* lipase activity revealed that methanol performs as a competitive inhibitor of the lipase enzyme^84^.

##### Effect of temperature on lipase activity

The effect of temperature on lipase activity of the two highly lipase-producing isolates *Kocuria flava* ASU5 (MT919305) and *Bacillus circulans* ASU11 (MT919306) was investigated at variable temperatures (10 °C to 60 °C), as shown in Fig. 7B. The results demonstrated that the lipase activity of bacterial isolates *Kocuria flava* ASU5 (MT919305) improved dramatically by increasing the temperature from 10 to 50 °C; after that, there was a significant drop in lipase activity. The optimal enzyme activity of bacterial isolate *Kocuria flava* ASU5 was recorded at 50 °C, whereas the results obtained for the lipase enzyme activity of bacterial isolate *Bacillus circulans* ASU11 (MT919306) revealed an increase in activity by increasing the temperature of the reaction from 10 to 60 °C. Data presented in Fig. 7C show that the highest lipase thermostable activity was recorded for *Kocuria flava* ASU5 compared to lipases from *Bacillus circulans* ASU11. Recently, there has been an increasing demand for thermotolerant lipases due to their promising applications in various industries. However, the majority of the microbial lipases exhibited the highest activity in the mesophilic range. The exploration for thermotolerant lipases has gained more attention as they have shown 70% to 100% of their activity in the temperature range of 50 °C to 70 °C^85^. Bora and Bora^86^, reported that lipases from *Bacillus* sp. revealed 90% enzyme activity at 60 °C for 1 h. as well as lipases from *Pseudomonas* sp. strain ZBC1 which exhibited maximum activity at 80 °C^87^.

##### Effect of pH on lipase activity

In this study, bacterial lipases of bacterial isolates *Kocuria flava* ASU5 (MT919305) and *Bacillus circulans* ASU11 (MT919306) were incubated in buffer systems with specific pH values (6, 7, 8, 9, and 10) to evaluate the impact of pH values on enzyme activity. The data showed that lipase activities increased with an increase in the pH value of the reaction mixture and that the optimal pH levels were 8 and 9 for lipase activities from bacterial strain *Kocuria flava* ASU5 and *Bacillus circulans* ASU11, respectively (Fig. 7D). Lipase activities dropped dramatically at pH 9. The maximum pH for the enzyme activity is located in the alkaline range. The majority of the microbial lipases have the highest activity in neutral or acidic reaction mixtures. But, alkaline lipases provide promising biotechnological applications in many prospective green chemistry industries. Bacterial lipases from *Bacillus thermoleovorans* and *Bacillus stearothermophilus* showed high stability in the alkaline pH range of 8 to 10^88^. From one-factor-at-a-time optimizing results, *Kocuria flava* ASU5 lipases showed the highest tolerance for methanol and thermostability as well as alkaline stability compared with *Bacillus circulans* ASU11 lipases. Consequently, *Kocuria flava* ASU5 was selected for optimizing lipase catalytic activity by central composite design (CCD) in addition to biodiesel production of cooking oil waste through lipase transesterification and esterification process under the optimized conditions.

#### Central composite design fitting and analysis of response surface

**Figure 8:**
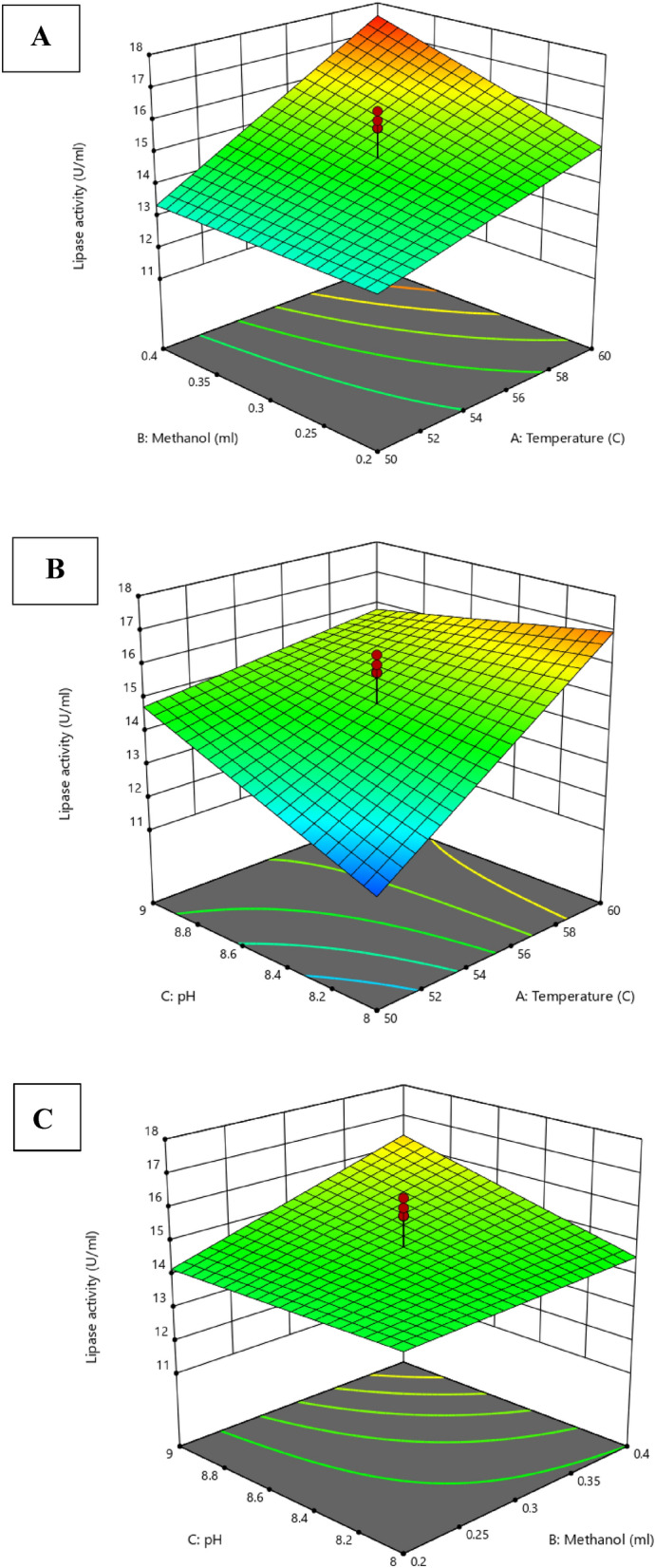
Three-dimensional plots of lipase catalytic activities by *Kocuria flava* ASU5 (MT919305) display the impacts of reaction mixture conditions: (**A**) interaction of Temperature/Methanol; (**B**) interaction of Temperature/pH; and (**C**) interaction of Methanol/pH.

The optimization of lipase catalytic activities and the interaction of the reaction mixture conditions were investigated using Response Surface Methodology (RSM). The optimal variables yielding high lipase activity for bacterial lipase ASU5 were selected and optimized even further using Central Composite Design (CCD). Each reaction condition factor was assayed at the coded levels (high and low); the obtained design for the factors at different levels is listed in Table (4). Statistical analysis of the model was stated by Fisher’s test value (F-value), and the results of the tested parameters and ANOVA analysis were recorded as shown in Tables 4 and 5. The F-value (ratio of mean square regression and mean square residual corresponding to the real error) of the proposed model is 8.398, indicating that the model statistics are significant. Moreover, the lack of fit value of the model is 2.96, which indicates that it is not significant and the pure error is 0.468769. The ANOVA analysis data revealed that among the tested variables, A (Temperature) and C (pH value) of the reaction conditions were found to be non-significant whereas B (Methanol) exhibited significant impacts. On the other hand, the interactions of AB (Temperature/Methanol) showed significant effects, whereas AC (Temperature/pH) and BC (pH/Methanol) revealed no significance. Three-dimensional curves and the coded model were created to investigate the interaction of reaction conditions to define the optimal conditions for maximizing the catalytic activities of lipases (Fig. 8). The 3D analysis revealed that optimal reaction conditions for optimizing the catalytic activities of bacterial lipase were: temperature 60 ^○^C; and methanol 0.4 (mL) at reaction mixture pH 8. The optimization of reaction conditions (depending on the classical method) only takes into consideration a single parameter effect, while all the other factors are interacting and working together at a fixed level. Therefore, statistically designed experimental models have been used to successfully explicate the interaction of different factors of the reactions and reduce the error in defining the impacts of reaction conditions^89^; this improves the process efficiency^90^. Lv et al.^91^ studied the impact of reaction conditions by using RSM; this involved examination of the soybean oil/methanol molar ratio, water content, free lipase amount, temperature, and reaction time on lipase catalytic activities and biodiesel production. Their results revealed the optimal conditions for lipase activities and the transesterification process were lipase load 5%, soybean oil/methanol molar ratio 1:7, water content 14%, temperature 38 °C, and reaction time 26 h. The maximum biodiesel yield (92.4 ± 0.8%) was obtained under optimal conditions.

**Table 4:** Reaction condition variables assayed for the experimental design of the quadratic model.

Run	Tested variables		
	Temperature ^○^C	Methanol ml	pH
1	55.0	0.30	8.5
2	50.0	0.20	8.0
3	55.0	0.13	8.5
4	50.0	0.40	9.0
5	55.0	0.30	9.3
6	55.0	0.30	8.5
7	55.0	0.30	8.5
8	60.0	0.20	8.0
9	55.0	0.30	8.5
10	55.0	0.45	8.5
11	55.0	0.30	8.5
12	60.0	0.20	9.0
13	63.4	0.30	8.5
14	60.0	0.40	8.0
15	55.0	0.30	8.5
16	60.0	0.40	9.0
17	46.6	0.30	8.5
18	50.0	0.20	9.0
19	55.0	0.30	7.7
20	50.0	0.40	8.0

**Table 5:** ANOVA analysis of the obtained results of lipase catalytic activities.

	Sum of squares	Degrees of freedom	Mean square	F-value	p-value	Significance
Model	52.062	6	8.677	8.398	0.0007	Significant
A-temperature	31.894	1	31.894	30.869	9.289E-05	Not significant
B-methanol	5.080	1	5.080	4.917	0.045	Significant
C-pH	2.501	1	2.500	2.420	0.144	Not significant
AB	2.667	1	2.667	2.582	0.132	Not significant
AC	8.013	1	8.013	7.755	0.015	Significant
BC	1.906	1	1.906	1.845	0.197	Not significant
Residual	13.432	13	1.033			
Lack of fit	11.088	8	1.386	2.957	0.124	Not significant
Pure error	2.344	5	0.469			
Cor total	65.493	19

### Biodiesel production by lipase transesterification and esterification process from cooking oil

**Figure 9:**
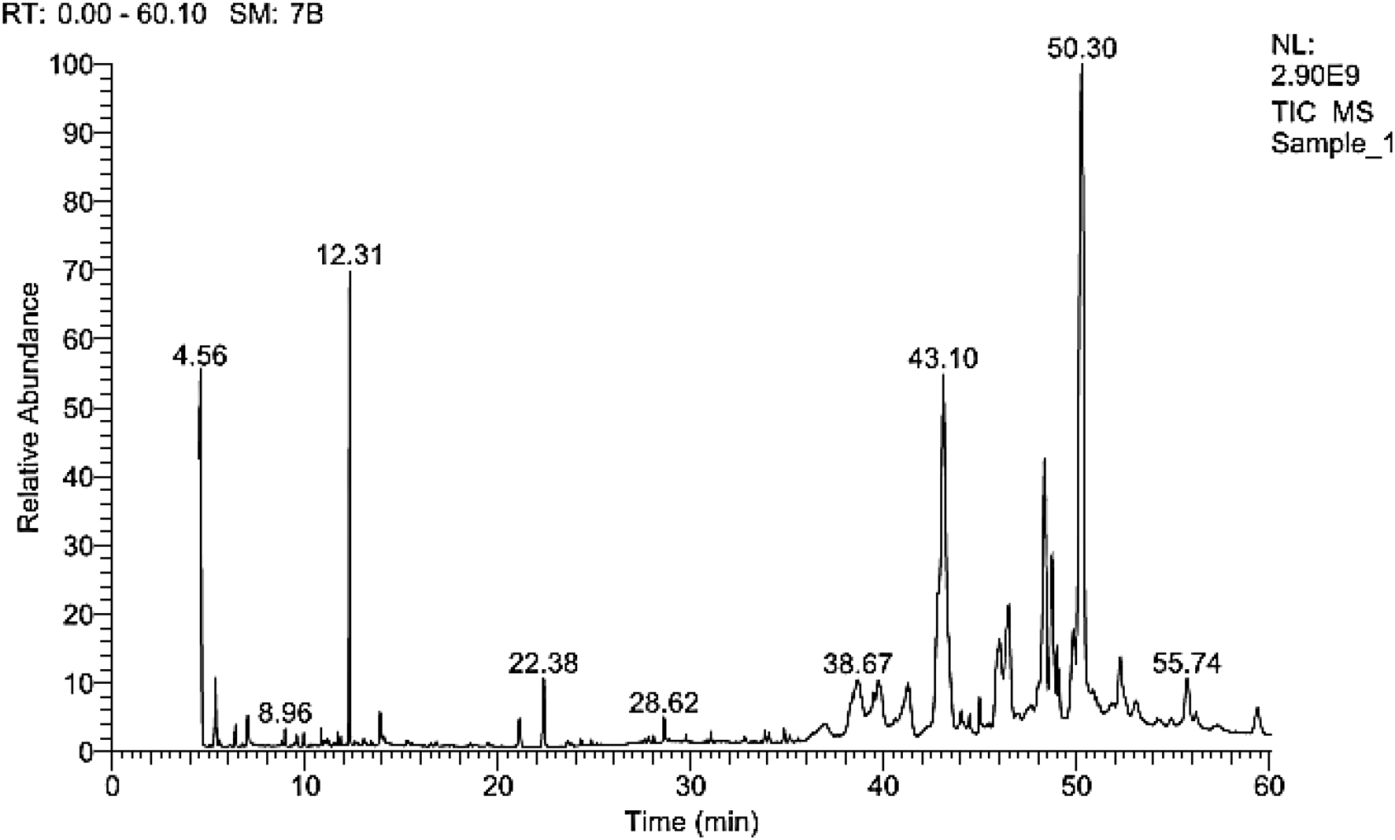
GC/MS profile of fatty acid methyl esters (biodiesel) produced from the enzymatic transesterification and enzymatic esterification process of cooking oil waste.

The highly applicable thermostable and methanol-tolerant lipase from *Kocuria flava* ASU5 (MT919305) was selected for biodiesel synthesis by use of the lipase transesterification and esterification process. Data obtained from the GC/MS analysis of biodiesel produced from the lipase transesterification and esterification of cooking oil waste under optimal conditions showed the highest activity for the transesterification and esterification of cooking oil waste (83.08% FAMEs), predicting the prospective applicability of bacterial lipases as a promising biocatalyst for the transesterification and esterification process due to lipases exhibiting a higher thermostability and methanol tolerance (Fig. 9). Interestingly, Abd-Alla et al.^76^ reported that free or immobilized cells of lipase-producing *Bacillus firmus* showed a high conversion (78% FAME) for converting fungal lipids into biodiesel. Cervero et al.^62^ reached 48% biodiesel by use of the one-step methanol addition method during the lipase esterification process, whereas 90% of the biodiesel production was obtained through the use of the three-step addition method. Furthermore, Xie and Wang^32^ stated that biodiesel yield from soybean oil using immobilized lipase reported that 86% was attained at a reaction temperature of 35 °C for 24 h with a biodiesel yield of 92.8% was recorded using a three-step methanol addition at 40 °C using *Candida rugosa* lipase bound on the Fe_3_O_4_ magnetic nanocomposite^92^. The lipase-catalyzed bioconversion of waste oil into biodiesel by thermostable and methanol-tolerant, bacteria-free lipases is considered one of the most promising technologies for the production of applicable, competitively priced, renewable, and eco-friendly alternative fuels.

### Fourier-transform infrared spectroscopy (FTIR) analysis

**Figure 10:**
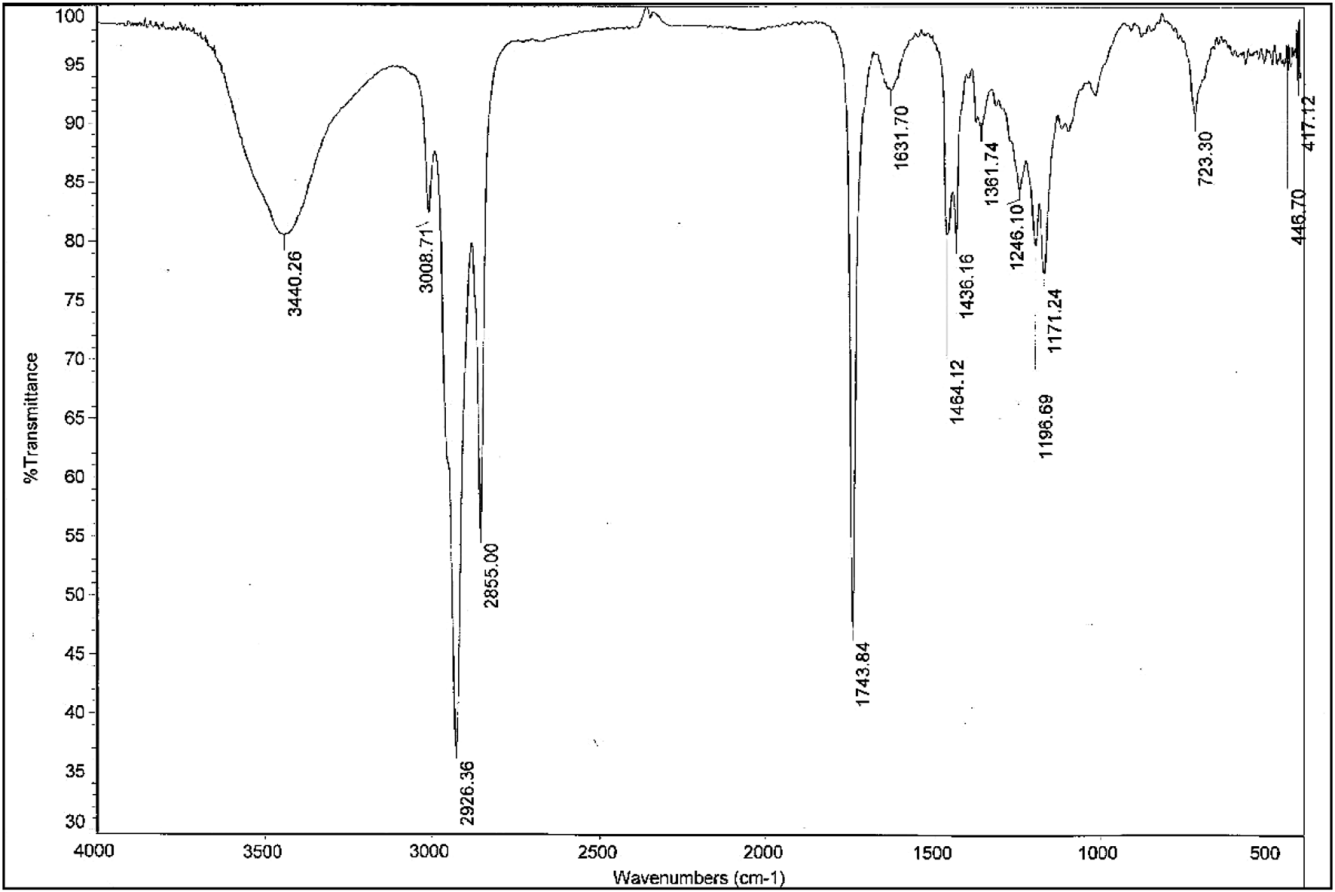
FTIR analysis of fatty acid methyl esters (biodiesel) produced from the enzymatic transesterification and enzymatic esterification process of cooking oil waste.

The FTIR methodology allows for the evaluation of the catalyzed reactions during transesterification and the hydrolysis process by lipases. This approach also reveals many advantages over the other traditional analytical approaches: It is simple, it offers rapid detection, it is a low-cost option, it is accurate, and there is no need for large amounts of samples or sample preparation^93^. This technique shows great potential for analyzing FAME content in biodiesel/diesel fuel, as reported in the literature^93^. From FTIR analysis presented in Fig. 10, our results revealed the presence of enormous functional groups which were well-fitted with other findings reported from the analysis of biodiesel and commercial diesel^93^. The data in Table 6 show the presence of absorption at 3440 cm^−1^, indicating stretching vibrations of the hydroxyl group (OH) and absorption at 3008 cm^−1^ revealed a stretching vibration (C–H bond). The presence of other peaks confirmed the products of the lipase transesterification and esterification process (FAME) as the absorption in the wavelengths 2926 cm^−1^ and 2855 cm^−1^ indicated stretching vibrations of the C–H bond (the methylene group [CH_2_])^94^. Interestingly, the absorption peak at 1446 cm^−1^ was attributed to the stretching of −CH_3_ in the biodiesel spectrum^95^. There was also an intense absorption band estimated at 1743 cm^−1^, which is recognized for its absorption in the carbonyl group (C=O) band (1700 cm^−1^ to 1800 cm^−1^)^96^. This band is characteristic for FAME absorption, as esters have characteristic, very strong C=O absorption bands (saturated aliphatic esters)^94,97^. Furthermore, C–O stretching vibrations were attributed to the appearance of the absorption peak at 1196 cm^−1^, which is characteristic for biodiesel^98^ as well as the absorption bands, in which 1246 cm^−1^ and 1171 cm^−1^ indicate long-chain FAME^94,99^.

**Table 6:** FTIR Analysis of the functional group of FAME produced from cooking oil waste lipase transesterification and esterification process.

Wavenumber (cm^−1^)	Function groups
3440	Stretching vibrations (O–H) in H_2_O
3008	Stretching vibrations (C–H)
2926	CH_2_, CH_3_, aliphatic group of triglycerides
2855	
1743	C=O group of Ester
1464	–C–H (in CH_2_) bending
1436	=C–H (cis-) bending
1361	–C–H (CH_3_) bending
1246	–C–O stretching/–O–CH_2_–C
1196	–C–O

### Biodiesel energy content

The energy content of 1 mol of biodiesel (docosanoic acid methyl ester) produced from cooking oil waste by the enzymatic transesterification and enzymatic esterification process was − 12,628.5 kJ/mol, indicating the entire combustion reaction was exothermic.

## Materials and methods

### Oily waste collection

Cooking oil was collected from domestic and restaurant cooking waste and placed in sterilized glass bottles; they were immediately transferred to and kept at 4 ^○^C in the laboratory. The composition of cooking oil waste was determined using GC/Ms (Thermo Scientific, Model: DPC-Direct Probe Controller (DPC-20451), USA; at the Chemistry Department, Faculty of Science, Assiut University).

### Isolation of lipase-producing bacteria from cooking oil waste

Thermotolerant bacteria were isolated from cooking oil waste on a culture medium: peptone, 10 g/L; NaCl, 5 g/L; CaCl_2_, 1 g/L; filtrated cooking oil waste (using Seitz filter 0.45 µM), 10 mL; and agar, 15 g/L. One mL of collected cooking oil waste was dispersed on the surface of the lipase detection medium and incubated at 50 ^○^C for 5 days. Positive lipase-producing isolates forming a white precipitate around the grown colonies were picked, subcultured on nutrient agar medium, and kept at 4 ^○^C for further experiments.

### Screening for lipase production

Thermotolerant bacterial isolates were grown aerobically in a glass bottle containing 30 mL of a liquid medium containing (g/L): peptone, 15; yeast extract, 5; NaCl, 2; MgSO_4_, 0.4; KH_2_PO_4_, 0.3; K_2_HPO_4_, 0.3; and filtrated cooking oil waste, 10 mL for lipase induction^100^. Bacterial cultures (three replicates) were incubated for 48 h under shaking conditions (120 rpm) at 50 ^○^C. Lipase enzymes in the culture broth were collected by centrifugation for 10 min at 3540×*g* to remove bacterial cells. The supernatant was then collected for lipase assay.

Lipase activity was determined spectrophotometrically by p-nitrophenyl palmitate (pNPP), according to the method of Tripathi et al.^101^. The enzyme reaction mixture was composed of 0.1 mL culture supernatant and 0.9 mL freshly prepared substrate solution (0.1 mM pNPP dissolved in 9 mL isopropanol and 0.8 mL of 100 mM phosphate buffer [pH 7]). This mixture was incubated for 30 min at 45 ^○^C. The absorbance was recorded at 410 nm and one lipase unit (IU) was defined as the amount of enzyme that liberated 1 µM p-nitrophenol per min under the standard assay condition. The standard curve was prepared using para-nitrophenol (0.4 µmol to 4 µmol). Each experiment was conducted with three replicates. The lipase-specific activity was defined as the number of lipase units per mg extracellular protein. The total extracellular protein was measured in the culture supernatant as described by Lowry et al.^102^, and a standard curve was prepared using bovine serum albumin.

### Screening for methanol-tolerant bacterial isolates

The highly lipase-producing bacterial isolates were selected to investigate their tolerance for methanol in the culture medium. Bacterial isolates were grown in a culture medium containing (g/L) peptone, 15; yeast extract, 5; NaCl, 2; MgSO_4_, 0.4; KH_2_PO_4_, 0.3; K_2_HPO_4_, 0.3, and filtrated cooking oil waste, 10 mL. After sterilization, the culture medium was supplemented with 10 mL/L filter-sterilized methanol. The bacterial inoculated cultures were incubated for 5 days; after that, lipase activity was assayed as previously described.

### Phenotypic and genotypic identification of highly lipase-producing bacterial isolates

The highly lipase-producing and methanol-tolerant isolates were identified based on microscopic and macroscopic characteristics and biochemical tests (Gram stain, oxidase reaction, nitrate reductase, catalase and urease test, utilization of carbohydrates, gelatin, starch and casein hydrolysis, Indole and H_2_S production and esculine hydrolyzation). The identity of isolates was confirmed at the genetic level by analyzing the partial 16S rRNA gene sequence using 27F and 1492R primers^67^. The obtained sequences of the 16S rRNA of bacterial strains were analyzed at the NCBI website: http://www.ncbi.nlm.nih.gov/BLAST/T using BLAST search program for assessing the DNA similarity degree.

### Optimization of organic solvent and alkaline stability and thermostability of bacterial lipases

The optimization procedures of lipase activities by the selected, highly lipase-producing strains were performed in two experimental stages.

#### Optimization of organic solvent stability and thermostability of bacterial lipases using the one-factor-at-a-time method

Various factors, including different organic solvents, organic solvent concentrations, different temperatures, and various pH values were tested on lipase enzyme activity to determine the effective factors for the highest catalytic enzyme activity in the reaction mixture.

##### Organic solvent stability of bacterial lipases

The effect of different acyl acceptors on enzyme activity of the highly lipase-producing and methanol-tolerant strains was investigated. The reaction mixture contained 0.9 mL of the substrate solution, 0.1 mL of lipase crude enzyme (bacterial supernatant), 0.1 mL different acyl acceptor (methanol, ethanol, propanol, butanol, and ethyl acetate), and 0.8 mL of 100 mM phosphate buffer (pH 7). As well the impact of different concentrations of acyl acceptor was investigated on lipase relative activity.

##### Effects of different temperatures and the thermostability of bacterial lipases

Different incubation temperatures were assayed on the enzyme activity of the highly lipase-producing and methanol-tolerant strains. The reaction mixture contained 0.9 mL of the substrate solution; 0.1 mL of lipase crude enzyme was incubated for 30 min at different temperatures (10 ^○^C to 60 ^○^C), the optimum temperature for enzyme activity was determined. The residual lipase activity was further estimated.

##### Effect of pH on lipase activity and stability

The activities of the bacterial lipases were assayed in various buffer systems (100 mM) with different pH values (6.0 to 10.0) at the optimal temperature for 3 h. The enzyme activity was measured according to the pNPP method described above.

#### Optimization of organic solvent, thermal, and alkaline stability of bacterial lipases by central composite design

The central composite design was used to study the interaction of the effective components of the reaction mixture conditions, which resulted from the one-factor-at-a-time method, for optimizing the process of the highest lipase catalytic activity from *Kocuria flava* ASU5 (MT919305). In this study, the organic solvent stability and thermostability of *Kocuria flava* ASU5 lipases were screened using the statistical software package Design-Expert (V12); StatEase, USA. Based on the central composite design, each factor was studied at two levels: low and high.

### Biodiesel synthesis by free cell-*Kocuria flava* ASU5 lipase transesterification and esterification process

#### Enzymatic transesterification and enzymatic esterification process

The organic solvent and alkaline stable and thermostable lipases produced from bacterial strain *Kocuria flava* ASU5 (MT919305) were selected for determining the potentiality of bacterial lipases under optimized reaction mixture conditions on the transesterification and esterification process for biodiesel (fatty acid methyl esters [FAMEs]) synthesis from cooking oil waste. The transesterification and esterification reaction assay was performed in 5 mL stopper glass vials containing cooking oil waste and methanol (1:2 oil/alcohol molar ratios), 1 mL bacterial supernatant enzyme (15 U/mL), and 1 mL buffer solution (100 mM [pH 8]). The reaction was incubated at 60 ^○^C for 5 h; then, 1 mL aliquot from the transesterification and esterification mixture system was withdrawn and mixed with 900 µL of n-hexane and then centrifuged at 4868xg for 10 min. A 2 µL of the produced fatty acid methyl esters (top layer) were collected and analyzed using GC/MS (Thermo Scientific, Model: DPC-Direct Probe Controller (DPC-20451), USA; at the Chemistry Department, Faculty of Science, Assiut University) to quantify the biodiesel yield (% FAME). A capillary column TG-5MS with a dimension of 30 m, 0.25 mm i.d., 1 µm film thicknesses was used for the separation of fatty acid esters. The oven temperature was initially maintained at 80 °C for 5 min, increased at 10 °C /min to 150 °C for 10 min, increased to 200 °C at ramp rate 10 °C/min (hold for 10 min) and finally the temperature was increased to 250 °C with ramp rate 5 °C/min, (hold time for 13 min). The split flow was 10 mL/min, and helium was used as carrier gas at a flow rate of 0.5 mL/min. Injector and detector temperatures were 250 °C and 300 °C, respectively. The % FAME (biodiesel) yield was determined by comparing the peak area of internal standard methyl esters at the particular retention time.

### Fourier-transform infrared spectroscopy (FTIR) analysis of the produced FAME product

The extracted FAME was analyzed using the KBr pressed disk technique (Thermo Scientific Nicolet iS10 FT-IR Spectrometer, USA) in the Chemistry Department of the Faculty of Science at Assiut University.

### Calculations of the gross energy content of the produced biodiesel

The total energy content of FAME produced by the transesterification and esterification process was determined based on the most prevalent fatty acid methyl ester (Docosanoic acid methyl ester). The energy of combustion could be calculated from the energy of bonds from the following Eqs. (1 and 2):

1 $${\text{C}}_{{{\text{23}}}} {\text{H}}_{{{\text{46}}}} {\text{O}}_{{\text{2}}} + {\text{ 67 1}}/{\text{2O}}_{{\text{2}}} \to {\text{ 23 H}}_{{\text{2}}} {\text{O }} + {\text{ 23 CO}}_{{\text{2}}} + {\text{ }}\left( {{\text{12628}}.{\text{5 kJ}}/{\text{mol}}} \right)$$

2 $${\text{H }} = {\text{ }}\sum {\text{BE }}\left( {{\text{bonds broken}}} \right){\text{ }} - {\text{ }}\sum {\text{BE }}\left( {{\text{bonds formed}}} \right)$$

In which BE is the bond energies

∆H = (28,703 + 16,582.5) − (36,754 + 21,160).

∆H = (45285.5 − 57914)

∆H = − 12628.5 kJ/mol

## Conclusions and future research trends

In this study, lipases from bacterial isolate *Kocuria flava* ASU5 (MT919305) exhibited the highest thermal and methanol tolerance (98.4% relative activity) and they showed considerable stability in an alkaline reaction mixture, so they were selected for enhancing the catalytic activity of lipase using RSM revealing the best and most efficient activity of the reaction system was noted at 60 ^○^C, oil/methanol molar ratios (1:2) and pH value of 8. The biodiesel production of 83.08% with an energy content of 12,628.5 kJ/mol was obtained from cooking oil. So, the current paper may enhance the feasibility of cleanly applicable technology for biodiesel production using cell-free, methanol- and thermo-tolerant lipases in batch reactors for the transesterification/esterification process of cooked oil waste. However, the lipase-mediated industrial transesterification processes are handicapped by some drawbacks concerning enzyme recovery and recyclability. To overwhelm these obstacles, prospective multifarious research attempts are required to encapsulate lipases on suitable carriers such as biological nanocomposites for the enhancement of lipase enzyme activities and consequently enzymatic transesterification and enzymatic esterification tactics. As well, the combination of enzymatic biocatalysts with other heterogeneous chemical catalysts or through using energy safely and high frequency piezoelectric ultrasonic reactors may enhance the economic feasibility of biodiesel technology. In addition to investigating the sustainability features of the obtained data using advanced sustainability assessment tools such as life cycle assessment, exergy and their consolidated combination of soft computing systems for improving the operating conditions may provide a promising fuel technology for prospective industrial bioenergy technologies.

## Abbreviations

16S rRNA: Small subunit ribosomal ribonucleic acid
3D: Three-dimensional
*B. circulans*: *Bacillus circulans*
*B. firmus*: *Bacillus firmus*
CCD: Central composite design
EtOH: Ethanol
FAAEs: Fatty acid alkyl esters
FAME: Fatty acid methyl ester
FFA: Free fatty acids
FTIR: Fourier-transform infrared spectroscopy
F-value: Fisher’s test value
GC/Ms: Gas chromatography-Mass spectroscopy
IU: International unit
*K. flava*: *Kocuria flava*
MeOH: Methanol
pNPP: p-Nitrophenyl palmitate
RSM: Response surface methodology

## List of chemical formulas and symbols with their chemical names

Bi_2_O_3_–La_2_O_3_: Bismuth(III) oxide–Lanthanum oxide
Ca(OCH_3_)_2_: Calcium methoxide
CaCl_2_: Calcium chloride
CaO: Calcium oxide
CH_3_ONa: Sodium methoxide
Fe(HSO_4_)_3_: Ferric hydrogensulfate
g-Al_2_O_3_/KI: Aluminum oxide/Potassium iodide
H_2_SO_4_: Sulfuric acid
H_3_PO_4_: Phosphoric acid
HCl: Hydrochloric acid
KF: Potassium fluoride
KOH: Potassium hydroxide
NaCl: Sodium chloride
NaNO_3_: Sodium nitrate
NaOH: Sodium hydroxide
ZrO_2_: Zirconium dioxide

## References

[1] Goldemberg J (2007). Ethanol for a sustainable energy future. Science.

[2] Hassan EA, Abd-Alla MH, Zohri AA, Ragaey MM, Ali SM (2019). Production of butanol and polyhydroxyalkanoate from industrial waste by *Clostridium beijerinckii* ASU10. Int. J. Energy Res..

[3] Panahi HKS, Dehhaghi M, Kinder JE, Ezeji TC (2019). A review on green liquid fuels for the transportation sector: A prospect of microbial solutions to climate change. Biofuel. Res. J..

[4] Hawkes FR, Dinsdale R, Hawkes DL, Hussy I (2002). Sustainable fermentative hydrogen production: Challenges for process optimisation. Int. J. Hydrog. Energy.

[5] Yaakob Z, Mohammad M, Alherbawi M, Alam Z, Sopian K (2013). Overview of the production of biodiesel from Waste cooking oil. Renew Sustain Energy Rev.

[6] Tabatabaei M, Aghbashlo M, Dehhaghi M, Panahi HKS, Mollahosseini A, Hosseini M, Soufiyan MM (2019). Reactor technologies for biodiesel production and processing: A review. Prog. Energy Combust. Sci..

[7] Islam A, Taufiq-Yap YH, Chan ES, Moniruzzaman M, Islam S, Nabi MN (2014). Advances in solid-catalytic and non-catalytic technologies for biodiesel production. Energy Convers. Manag.

[8] Teo SH, Islam A, Ng FL, Taufiq-Yap YH (2015). Biodiesel synthesis from photoautotrophic cultivated oleoginous microalgae using a sand dollar catalyst. RSC Adv..

[9] Xu G, Wu G-Y (2003). The investigation of blending properties of biodiesel and No 0 diesel fuel. J. Jiangsu Polytech. Uni..

[10] Alhassan FH, Yunus R, Rashid U, Sirat K, Islam A, Lee HV, Taufiq-Yap YH (2013). Production of biodiesel from mixed waste vegetable oils using Ferric hydrogen sulphate as an effective reusable heterogeneous solid acid catalyst. Appl. Catal. A. Gen.

[11] Islam A, Taufiq-Yap YH, Chu CM, Ravindra P, Chan ES (2013). Transesterification of palm oil using KF and NaNO_3_ catalysts supported on spherical millimetric γ-Al_2_O_3_. Renew. Energy.

[12] Nur ZS, Taufiq-Yap YH, Nizah MR, Teo SH, Syazwani ON, Islam A (2014). Production of biodiesel from palm oil using modified Malaysian natural dolomites. Energy Convers. Manag..

[13] Taufiq-Yap YH, Teo SH, Rashid U, Islam A, Hussien MZ, Lee KT (2014). Transesterification of *Jatropha curcas* crude oil to biodiesel on calcium lanthanum mixed oxide catalyst: Effect of stoichiometric composition. Energy Convers. Manag..

[14] Nizah MR, Taufiq-Yap YH, Rashid U, Teo SH, Nur ZS, Islam A (2014). Production of biodiesel from non-edible *Jatropha curcas* oil via transesterification using Bi_2_O_3_–La_2_O_3_ catalyst. Energy Convers. Manag..

[15] Teo SH, Islam A, Yusaf T, Taufiq-Yap YH (2014). Transesterification of *Nannochloropsis oculata* microalga's oil to biodiesel using calcium methoxide catalyst. Energy.

[16] Islam A, Taufiq-Yap YH, Ravindra P, Teo SH, Sivasangar S, Chan ES (2015). Biodiesel synthesis over millimetric g-Al_2_O_3_/KI catalyst. Energy.

[17] Theam KL, Islam A, Choo YM, Taufiq-Yap YH (2015). Biodiesel from low cost palm stearin using metal doped methoxide solid catalyst. Ind Crops Prod..

[18] Theam KL, Islam A, Lee HV, Taufiq-Yap YH (2015). Sucrose-derived catalytic biodiesel synthesis from low cost palm fatty acid distillate. Process. Saf. Environ. Prot..

[19] Syazwani ON, Teo SH, Islam A, Taufiq-Yap YH (2017). Transesterification activity and characterization of natural CaO derived from waste venus clam (*Tapes belcheri* S.) material for enhancement of biodiesel production. Process. Saf. Environ. Prot..

[20] Ibrahim SF, Asikin-Mijan N, Ibrahim ML, Abdulkareem-Alsultan G, Izham SM, Taufiq-Yap YH (2020). Sulfonated functionalization of carbon derived corncob residue via hydrothermal synthesis route for esterification of palm fatty acid distillate. Energy Convers. Manag..

[21] Abd Rahman NJ, Ramli A, Jumbri K, Uemura Y (2019). Tailoring the surface area and the acid–base properties of ZrO_2_ for biodiesel production from *Nannochloropsis* sp.. Sci. Rep..

[22] Teo SH, Islam A, Ng CH, Mansir N, Ma T, Choong ST, Taufiq-Yap YH (2018). Methoxy-functionalized mesostructured stable carbon catalysts for effective biodiesel production from non-edible feedstock. Chem. Eng. J..

[23] Teo SH, Islam A, Taufiq-Yap YH (2016). Algae derived biodiesel using nanocatalytic transesterification process. Chem. Eng. Res. Des..

[24] Abdulkareem-Alsultan G, Asikin-Mijan N, Mansir N, Lee HV, Zainal Z, Islam A, Taufiq-Yap YH (2019). Pyro-lytic de-oxygenation of waste cooking oil for green diesel production over Ag_2_O_3_-La_2_O_3_/AC nano-catalyst. J. Anal. Appl. Pyrolysis.

[25] Ibrahim ML, Khalil NN, Islam A, Rashid U, Ibrahim SF, Mashuri SIS, Taufiq-Yap YH (2020). Preparation of Na_2_O supported CNTs nanocatalyst for efficient biodiesel production from waste-oil. Energy Convers. Manag..

[26] Teo SH, Islam A, Masoumi HRF, Taufiq-Yap YH, Janaun J, Chan ES (2017). Effective synthesis of biodiesel from *Jatropha curcas* oil using betaine assisted nanoparticle heterogeneous catalyst from eggshell of *Gallus domesticus*. Renew. Energy.

[27] Zhao X, Qi F, Yuan C, Du W, Liu D (2015). Lipase-catalyzed process for biodiesel production: Enzyme immobilization, process simulation and optimization. Renew. Sustain. Energy Rev..

[28] Macario, A., Moliner, M., Diaz, U., Jorda, J., Corma, A., & Giordano, G. Biodiesel production by immobilized lipase on zeolites and related materials. in Studies in surface science and catalysis, 1011–1016 (Elsevier, 2008).

[29] Salis A, Pinna M, Monduzzi M, Solinas V (2005). Biodiesel production from triolein and short chain alcohols through biocatalysis. J. Biotechnol..

[30] Andrade TA, Errico M, Christensen KV (2017). Evaluation of reaction mechanisms and kinetic parameters for the transesterification of castor oil by liquid enzymes. Ind. Eng. Chem. Res..

[31] Ilmi M, Hommes A, Winkelman J, Hidayat C, Heeres H (2016). Kinetic studies on the transesterification of sunflower oil with 1-butanol catalyzed by *Rhizomucor miehei* lipase in a biphasic aqueous-organic system. Biochem. Eng. J..

[32] Xie W, Wang J (2014). Enzymatic production of biodiesel from soybean oil by using immobilized lipase on Fe_3_O_4_/poly (styrene-methacrylic acid) magnetic microsphere as a biocatalyst. Energ Fuel.

[33] Barbosa O, Ariza C, Ortiz C, Torres R (2010). Kinetic resolution of (R/S)-propranolol (1-isopropylamino-3-(1-naphtoxy)-2-propanolol) catalyzed by immobilized preparations of *Candida antarctica* lipase B (CAL-B). New Biotechnol..

[34] Xie W, Huang M (2018). Immobilization of *Candida rugosa* lipase onto graphene oxide Fe_3_O_4_ nanocomposite: Characterization and application for biodiesel production. Energy Convers. Manag..

[35] Xu Z, Wang S, Li Y, Wang M, Shi P, Huang X (2014). Covalent functionalization of graphene oxide with biocompatible poly (ethylene glycol) for delivery of paclitaxel. ACS Appl. Mater. Interfaces.

[36] Verma ML, Naebe M, Barrow CJ, Puri M (2013). Enzyme immobilisation on amino-functionalised multi-walled carbon nanotubes: structural and biocatalytic characterisation. PLoS ONE.

[37] Sharma RK, O'Neill CA, Ramos HA, Thapa B, Barcelo-Bovea VC, Gaur K, Griebenow K (2019). *Candida rugosa* lipase nanoparticles as robust catalyst for biodiesel production in organic solvents. Biofuel Res. J..

[38] Freitas VOD, Matte CR, Poppe JK, Rodrigues RC, Ayub MA (2019). Ultrasound-assisted transesterification of soybean oil using combi-lipase biocatalysts. Braz. J. Chem. Eng..

[39] Özbek B, Ülgen KÖ (2000). The stability of enzymes after sonication. Process Biochem..

[40] Aghbashlo M, Tabatabaei M, Hosseinpour S (2018). On the exergoeconomic and exergoenvironmental evaluation and optimization of biodiesel synthesis from waste cooking oil (WCO) using a low power, high frequency ultrasonic reactor. Energy Convers. Manag..

[41] Aghbashlo M, Hosseinpour S, Tabatabaei M, Soufiyan MM (2019). Multi-objective exergetic and technical optimization of a piezoelectric ultrasonic reactor applied to synthesize biodiesel from waste cooking oil (WCO) using soft computing techniques. Fuel.

[42] Dossat V, Combes D, Marty A (2002). Lipase-catalysed transesterification of high oleic sunflower oil. Enzyme Microb. Technol..

[43] Dantas M, Conceição M, Fernandes V, Santos N, Rosenhaim R, Marques A, Santos I, Souza A (2007). Thermal and kinetic study of corn biodiesel obtained by the methanol and ethanol routes. J. Therm. Anal. Calorim..

[44] Shu Q, Zhang Q, Xu G, Nawaz Z, Wang D, Wang J (2009). Synthesis of biodiesel from cottonseed oil and methanol using a carbon-based solid acid catalyst. Fuel Process. Technol..

[45] Leung DY, Wu X, Leung M (2010). A review on biodiesel production using catalyzed transesterification. Appl. Energy.

[46] Fan, X., Niehus, X., & Sandoval, G. Lipases as biocatalyst for biodiesel production. in Lipases and phospholipases, 471–483 (Springer, 2012). 10.1007/978-1-61779-600-5_27 22426735

[47] Kolet M, Zerbib D, Nakonechny F, Nisnevitch M (2020). Production of biodiesel from brown grease. Catalysts.

[48] Panchal BM, Deshmukh SA, Sharma MR (2017). Production and kinetic transesterification of biodiesel from yellow grease with dimethyl carbonate using methanesulfonic acid as a catalyst. Environ. Prog. Sustain. Energy..

[49] Teo SH, Islam A, Chan ES, Choong ST, Alharthi NH, Taufiq-Yap YH, Awual MR (2019). Efficient biodiesel production from *Jatropha curcus* using CaSO_4_/Fe_2_O_3_-SiO_2_ core-shell magnetic nanoparticles. J. Clean. Prod..

[50] Gargari MH, Sadrameli S (2018). Investigating continuous biodiesel production from linseed oil in the presence of a Co-solvent and a heterogeneous based catalyst in a packed bed reactor. Energy.

[51] Karmakar B, Dhawane SH, Halder G (2018). Optimization of biodiesel production from castor oil by Taguchi design. J. Environ. Chem. Eng..

[52] Sarno M, Iuliano M (2019). Biodiesel production from waste cooking oil. Green Process. Synth..

[53] Jaeger K-E, Eggert T (2002). Lipases for biotechnology. Curr. Opin. Biotechnol..

[54] Mendes DB, Da Silva F, Guarda PM, Almeida AFD, de Oliveira D, Morais PBD, Guarda EA (2019). Lipolytic enzymes with hydrolytic and esterification activities produced by filamentous fungi isolated from decomposition leaves in an aquatic environment. Enzyme Res..

[55] Thangaraj B, Solomon PR, Muniyandi B, Ranganathan S, Lin L (2019). Catalysis in biodiesel production: A review. Clean. Energy..

[56] Yeoman CJ, Han Y, Dodd D, Schroeder CM, Mackie RI, Cann IKO (2010). Thermostable enzymes as biocatalysts in the biofuel industry. Adv. Appl. Microbiol..

[57] Avhad, M.R., & Marchetti, J.M. Uses of enzymes for biodiesel production. in Advanced Bioprocessing for Alternative Fuels, Biobased Chemicals, and Bioproducts, 135–152 (Elsevier, 2019).

[58] Rahman R, Chin J, Salleh A, Basri M (2003). Cloning and expression of a novel lipase gene from *Bacillus sphaericus* 205y. Mol. Genet. Genom..

[59] Hasan F, Shah AA, Hameed A (2006). Industrial applications of microbial lipases. Enzyme Microb. Technol..

[60] Wang X, Qin X, Li D, Yang B, Wang Y (2017). One-step synthesis of high-yield biodiesel from waste cooking oils by a novel and highly methanol-tolerant immobilized lipase. Bioresour. Technol..

[61] Antczak MS, Kubiak A, Antczak T, Bielecki S (2009). Enzymatic biodiesel synthesis–key factors affecting efficiency of the process. Renew. Energy.

[62] Cervero J, Alvarez J, Luque S (2014). Novozym 435-catalyzed synthesis of fatty acid ethyl esters from soybean oil for biodiesel production. Biomass Bioenergy.

[63] Cubides-Roman DC, Pérez VH, de Castro HF, Orrego CE, Giraldo OH, Silveira EG, David GF (2017). Ethyl esters (biodiesel) production by *Pseudomonas fluorescens* lipase immobilized on chitosan with magnetic properties in a bioreactor assisted by electromagnetic field. Fuel.

[64] Li K, Wang J, He Y, Cui G, Abdulrazaq MA, Yan Y (2018). Enhancing enzyme activity and enantioselectivity of *Burkholderia cepacia* lipase via immobilization on melamine-glutaraldehyde dendrimer modified magnetic nanoparticles. Chem. Eng..

[65] Kim SH, Kim S-J, Park S, Kim HK (2013). Biodiesel production using cross-linked *Staphylococcus haemolyticus* lipase immobilized on solid polymeric carriers. J. Mol. Catal. B Enzym..

[66] Shah S, Sharma S, Gupta M (2004). Biodiesel preparation by lipase-catalyzed transesterification of Jatropha oil. Energ Fuel.

[67] Ji Q, Wang B, Tan J, Zhu L, Li L (2016). Immobilized multienzymatic systems for catalysis of cascade reactions. Process. Biochem..

[68] Handayani N, Wahyuningrum D, Zulfikar MA, Nurbaiti S, Radiman CL (2016). The synthesis of biodiesel catalyzed by *Mucor miehei* lipase immobilized onto aminated polyethersulfone membranes. Bioresour. Bioprocess..

[69] Touqeer T, Mumtaz MW, Mukhtar H, Irfan A, Akram S, Shabbir A, Rashid U, Nehdi IA, Choong TSY (2020). Fe_3_O_4_-PDA-lipase as surface functionalized nano biocatalyst for the production of biodiesel using waste cooking oil as feedstock: characterization and process optimization. Energies.

[70] Duarte SH, del Peso Hernández GL, Canet A, Benaiges MD, Maugeri F, Valero F (2015). Enzymatic biodiesel synthesis from yeast oil using immobilized recombinant *Rhizopus oryzae* lipase. Bioresour. Technol..

[71] Lara PV, Park EY (2004). Potential application of waste activated bleaching earth on the production of fatty acid alkyl esters using *Candida cylindracea* lipase in organic solvent system. Enzyme Microb. Technol..

[72] Lee JH, Kim SB, Yoo HY, Lee JH, Han SO, Park C, Kim SW (2013). Co-immobilization of *Candida rugosa* and *Rhyzopus* oryzae lipases and biodiesel production. Korean J. Chem. Eng..

[73] Najjar A, Robert S, Guérin C, Violet-Asther M, Carrière F (2011). Quantitative study of lipase secretion, extracellular lipolysis, and lipid storage in the yeast *Yarrowia lipolytica* grown in the presence of olive oil: Analogies with lipolysis in humans. Appl. Microbiol. Biotechnol..

[74] Zhou G, Luo X, Tang Y, Zhang L, Yang Q, Qiu Y, Fang C (2008). Kocuria flava sp nov and Kocuria turfanensis sp nov, airborne actinobacteria isolated from Xinjiang, China. Int. J. Syst. Evol. Microbiol..

[75] Priest, F.G. Systematics and ecology of *Bacillus*. *Bacillus subtilis* and other gram‐positive bacteria: Biochemistry, physiology, and molecular genetics, 1–16 (1993).

[76] Abd-Alla MH, Bagy MMK, Morsy FM, Hassan EA (2015). Improvement of fungal lipids esterification process by bacterial lipase for biodiesel synthesis. Fuel.

[77] Faber K (1992). Biotransformations in Organic Chemistry.

[78] Santambrogio C, Sasso F, Natalello A, Brocca S, Grandori R, Doglia SM, Lotti M (2013). Effects of methanol on a methanol-tolerant bacterial lipase. Appl. Microbiol. Biotechnol..

[79] Adamczak M, Bornscheuer UT, Bednarski W (2009). The application of biotechnological methods for the synthesis of biodiesel. Eur. J. Lipid Sci. Tech..

[80] Robles-Medina A, González-Moreno P, Esteban-Cerdán L, Molina-Grima E (2009). Biocatalysis: towards ever greener biodiesel production. Biotechnol. Adv..

[81] Coggon R, Vasudevan PT, Sanchez F (2007). Enzymatic transesterification of olive oil and its precursors. Biocatal. Biotransform..

[82] Bozbas K (2008). Biodiesel as an alternative motor fuel: Production and policies in the European Union. Renew. Sustain. Energy Rev..

[83] Aghababaie M, Beheshti M, Razmjou A, Bordbar A-K (2017). Enzymatic biodiesel production from crude *Eruca sativa* oil using *Candida rugosa* lipase in a solvent-free system using response surface methodology. Biofuel..

[84] Fjerbaek L, Christensen KV, Norddahl B (2009). A review of the current state of biodiesel production using enzymatic transesterification. Biotechnol. Bioeng..

[85] Gohel H, Ghosh S, Bragazna V (2013). Production, purification and immobilization of extracellular lipases from thermophilic *Bacillus subtilis* XRF 11 and *Bacillus licheniformis* XRF 12 for production of alkyl esters. Int. J. Life Sci..

[86] Bora L, Bora M (2012). Optimization of extracellular thermophilic highly alkaline lipase from thermophilic *Bacillus* sp. isolated from Hot spring of Arunachal Pradesh, India. Braz. J. Microbiol..

[87] Xing C, You-guang P, Chen-xi Z, Zhi-lin R, Ming-xing J, Yan C, Bu-chang Z (2013). Screening of thermophilic neutral lipase-producing *Pseudomonas* sp. ZBC1 and improving its enzymatic activity. Afr. J. Biotechnol..

[88] Prazeres JND, Cruz JAB, Pastore GM (2006). Characterization of alkaline lipase from *Fusarium oxysporum* and the effect of different surfactants and detergents on the enzyme activity. Braz. J. Microbiol..

[89] Lakshmi M, Sridevi V, Rao MN, Swamy A (2011). Optimization of phenol degradation from *Pseudomonas aeruginosa* (NCIM 2074) using response surface methodology. Int. J. Res. Pharm. Chem..

[90] Ruchi G, Anshu G, Khare S (2008). Lipase from solvent tolerant *Pseudomonas aeruginosa* strain: Production optimization by response surface methodology and application. Bioresour. Technol..

[91] Lv Y, Sun S, Liu J (2019). Biodiesel production catalyzed by a methanol-tolerant lipase A from *Candida antarctica* in the presence of excess water. ACS Omega.

[92] Xie W, Huang M (2020). Fabrication of immobilized *Candida rugosa* lipase on magnetic Fe_3_O_4_-poly (glycidyl methacrylate-co-methacrylic acid) composite as an efficient and recyclable biocatalyst for enzymatic production of biodiesel. Renew. Energy.

[93] Natalello A, Sasso F, Secundo F (2013). Enzymatic transesterification monitored by an easy-to-use Fourier transform infrared spectroscopy method. Biotechnol. J..

[94] Rabelo SN, Ferraz VP, Oliveira LS, Franca AS (2015). FTIR analysis for quantification of fatty acid methyl esters in biodiesel produced by microwave-assisted transesterification. Int. J. Environ. Sci. Dev..

[95] Soares IP, Rezende TF, Silva RC, Castro EVR, Fortes IC (2008). Multivariate calibration by variable selection for blends of raw soybean oil/biodiesel from different sources using Fourier transform infrared spectroscopy (FTIR) spectra data. Energy Fuel.

[96] Matwijczuk A, Górecki A, Kamiński D, Myśliwa-Kurdziel B, Fiedor L, Niewiadomy A, Karwasz GP, Gagoś M (2015). Influence of solvent polarizability on the keto-enol equilibrium in 4-[5-(naphthalen-1-ylmethyl)-1, 3, 4-thiadiazol-2-yl] benzene-1, 3-diol. J. Fluoresc..

[97] Aliske MA, Zagonel GF, Costa BJ, Veiga W, Saul CK (2007). Measurement of biodiesel concentration in a diesel oil mixture. Fuel.

[98] Siddiqui N, Ahmad A (2013). Infrared spectroscopic studies on edible and medicinal oils. Int. J. Sci. Environ. Technol..

[99] Oliveira JS, Montalvao R, Daher L, Suarez PA, Rubim JC (2006). Determination of methyl ester contents in biodiesel blends by FTIR-ATR and FTNIR spectroscopies. Talanta.

[100] Godfrey T, Reichelt J (1982). Industrial Enzymology: The Application of Enzymes in Industry.

[101] Tripathi R, Singh J, Kumarbharti R, Thakur IS (2014). Isolation, purification and characterization of lipase from *Microbacterium* sp. and its application in biodiesel production. Energy Procedia.

[102] Lowry OH, Rosebrough NJ, Farr AL, Randall RJ (1951). Protein measurement with the Folin phenol reagent. J. Biol. Chem..

